# Consequences of Converting Graded to Action Potentials upon Neural Information Coding and Energy Efficiency

**DOI:** 10.1371/journal.pcbi.1003439

**Published:** 2014-01-23

**Authors:** Biswa Sengupta, Simon Barry Laughlin, Jeremy Edward Niven

**Affiliations:** 1 Wellcome Trust Centre for Neuroimaging, University College London, London, United Kingdom; 2 Centre for Neuroscience, Indian Institute of Science, Bangalore, India; 3 Department of Zoology, University of Cambridge, Cambridge, United Kingdom; 4 School of Life Sciences and Centre for Computational Neuroscience and Robotics, University of Sussex, Falmer, Brighton, United Kingdom; Indiana University, United States of America

## Abstract

Information is encoded in neural circuits using both graded and action potentials, converting between them within single neurons and successive processing layers. This conversion is accompanied by information loss and a drop in energy efficiency. We investigate the biophysical causes of this loss of information and efficiency by comparing spiking neuron models, containing stochastic voltage-gated Na^+^ and K^+^ channels, with generator potential and graded potential models lacking voltage-gated Na^+^ channels. We identify three causes of information loss in the generator potential that are the by-product of action potential generation: (1) the voltage-gated Na^+^ channels necessary for action potential generation increase intrinsic noise and (2) introduce non-linearities, and (3) the finite duration of the action potential creates a ‘footprint’ in the generator potential that obscures incoming signals. These three processes reduce information rates by ∼50% in generator potentials, to ∼3 times that of spike trains. Both generator potentials and graded potentials consume almost an order of magnitude less energy per second than spike trains. Because of the lower information rates of generator potentials they are substantially less energy efficient than graded potentials. However, both are an order of magnitude more efficient than spike trains due to the higher energy costs and low information content of spikes, emphasizing that there is a two-fold cost of converting analogue to digital; information loss and cost inflation.

## Author Summary

As in electronics, many of the brain's neural circuits convert continuous time signals into a discrete-time binary code. Although some neurons use only graded voltage signals, most convert these signals into discrete-time action potentials. Yet the costs and benefits associated with such a switch in signalling mechanism are largely unexplored. We investigate why the conversion of graded potentials to action potentials is accompanied by substantial information loss and how this changes energy efficiency. Action potentials are generated by a large cohort of noisy Na^+^ channels. We show that this channel noise and the added non-linearity of Na^+^ channels destroy input information provided by graded generator potentials. Furthermore, action potentials themselves cause information loss due to their finite widths because the neuron is oblivious to the input that is arriving during an action potential. Consequently, neurons with high firing rates lose a large amount of the information in their inputs. The additional cost incurred by voltage-gated Na^+^ channels also means that action potentials can encode less information per unit energy, proving metabolically inefficient, and suggesting penalisation of high firing rates in the nervous system.

## Introduction

Information is encoded, processed and transmitted in neural circuits both as graded potentials (continuous, analogue) and action potentials (pulsatile, digital). Although sensory and chemical synaptic inputs to neurons are graded [1], in most neurons these are converted into a train of action potentials. This conversion overcomes the attenuation of graded signals that occurs as they are propagated over long distances within the nervous system [2], and may prevent noise accumulation in neural networks because pulsatile signals are restored at each successive processing stage [3], [4]. However, because spike trains use discrete pulses of finite precision they have a lower dimensionality than analogue voltage signals, reducing their signal entropy [4]. Consequently, spike trains can encode fewer states within a given time period than analogue voltage signals. This is borne out by experimental measurements that show the conversion of the graded generator potential into a spike train reduces the information rate [5]–[7]. Thus, non-spiking neurons that encode information as graded potentials typically have much higher information rates than spiking neurons [5],[8],[9].

A drop in the energy efficiency of information coding has also been suggested to accompany the conversion of graded to action potentials [3], [10]. Neuronal energy consumption is dominated by the influx/efflux of ions, which must be pumped back across the cell membrane by the Na^+^/K^+^ ATPase consuming ATP [3], [11], [12]. These ion movements can incur substantial energy costs even in graded potential neurons [3], [13]. However, the large Na^+^ influx during action potentials requires additional cellular energy to extrude, though the precise energy cost will vary among neuron types [11], [14]–[16].

Our aim is to identify the causes of the loss of information and energy efficiency when graded potentials are converted to action potentials. Although some causes of information loss in spiking neurons have been studied previously, such as channel noise [17]–[19] or dimensionality reduction [6], [20], in most cases their effects on information rates have not been quantified. We quantified both information rates and energy efficiency using single compartment models. We compared the information rates, energy consumptions and energy efficiencies of spike trains with those of the generator potentials that triggered the spike trains, and of the graded response produced in the absence of voltage-gated Na^+^ channels. We find that three previously unreported effects reduce the information rate and efficiency of the generator potential by 50%; namely the finite durations of action potentials, and the noise and nonlinearity introduced by voltage-gated ion channels. The effect of channel noise on spike timing reduces the information rate and efficiency by <10%. We conclude that the conversion of graded signals to “digital” action potentials imposes two penalties; spikes increase energy costs and both spike coding mechanisms and the spike code reduce information rates. As a result energy efficiency falls by well over 90%.

## Results

**Figure 1:**
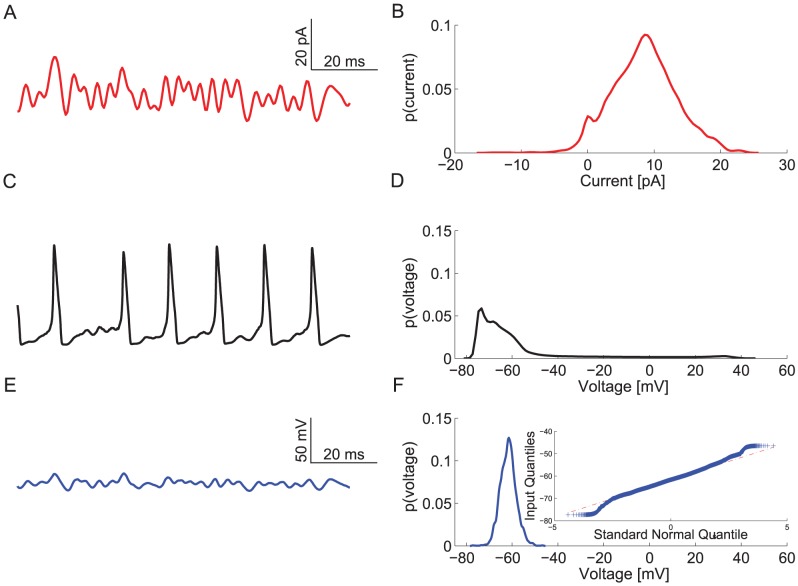
Voltage responses of spiking and graded potentials. A. The band-limited 300 Hz filtered Gaussian white noise current stimulus. B. The probably density function (PDF) of the current stimulus shown in A. C. A voltage response of the spiking neuron model to the current stimulus shown in A. D. The PDF of the spiking neuron model's voltage response. E. A voltage response of the graded neuron model to the current stimulus shown in A. F. The PDF of the graded neuron model's voltage response. (Inset) A QQ plot showing departures from a Gaussian distribution (dotted red-line) for the time-series shown in E.

We simulated the responses of a 100 µm^2^ single compartment model containing stochastic voltage-gated Na^+^ and K^+^ channels to a 300 Hz band-limited white-noise current stimulus to assess information coding in a spiking neuron model (see Methods) (Figure 1A,B). By altering the stimulus mean and standard deviation the model captured a wide range of neuronal activity patterns. Low mean, high standard deviation inputs produced voltage responses that resembled relay neurons, the activity of which is dominated by large post-synaptic potentials from relatively few pre-synaptic neurons, such as principle cells of the Medial Nucleus of the Trapezoid Body that receive synaptic inputs from the Calyx of Held [21]. High mean, low standard deviation inputs produced voltage responses that resembled those of integrator neurons, the activity of which is determined by a large number of small post-synaptic potentials, such as motor neurons [22].

By incorporating voltage-gated Na^+^ and K^+^ channels within the same compartment as a current input stimulus, we modelled the conversion of an analogue signal into a train of action potentials (APs or spikes), as would occur at the spike initiation zone of a neuron [23]. No extrinsic noise was added to the current stimulus in most of our simulations, consequently stochastic fluctuations of the voltage-gated ion channels were the only noise source. This stimulus produced small, sub-threshold graded fluctuations in membrane potential as well as action potentials approximately 100 mV in amplitude (Figure 1C). These transient 100 mV excursions to the peak voltage produced a skewed probability density function (PDF) of the membrane potential with a long tail (Figure 1D).

**Figure 2:**
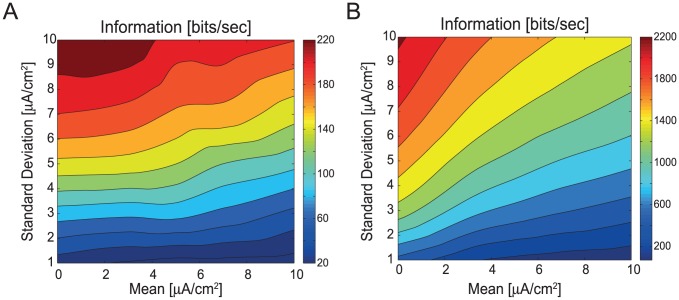
Information encoding in the spiking and graded neuron models. A. Information rates (bits s^−1^) of the spiking neuron model evoked by white noise current stimuli with different means and standard deviations. B. Information rates (bits s^−1^) of the graded neuron model evoked by the same white noise current stimuli as in A.

We compared the information encoded by the spiking neuron model with that encoded by an equivalent analogue model in response to the same white-noise current stimuli with varying mean amplitudes and standard deviations (Figure 1E,F). The analogue model lacked voltage-gated Na^+^ channels but was identical to the spiking neuron model in all other respects. In this model, current stimuli produced small, graded fluctuations in membrane potential with an approximately Gaussian PDF (Figure 1F). We extracted the power spectra of the signal and noise from these graded fluctuations and used them to calculate Shannon information rates [24], [25] (Methods). The rates at which spike trains coded information was calculated from the total entropy and noise entropy of the spikes using the direct method [26]. Both models, graded and spiking, encoded the most information when stimulated by low mean, high standard deviation currents and the least information with high mean, low standard deviation currents (Figure 2A,B). Thus, the information rate of both neuron models is critically dependent upon the statistics of the input stimulus.

The information encoded by the graded neuron model for each input stimulus was greater than that of the spiking neuron model (Figure 2A,B). The highest information rate attained by the spiking neuron model was 235 bits/s, whereas the graded neuron model attained information rates of 2240 bits/s. Thus, the graded neuron model encodes almost an order of magnitude more information per second than the spiking neuron model, reproducing experimentally observed differences between graded and spiking neurons [5]–[8].

**Figure 3:**
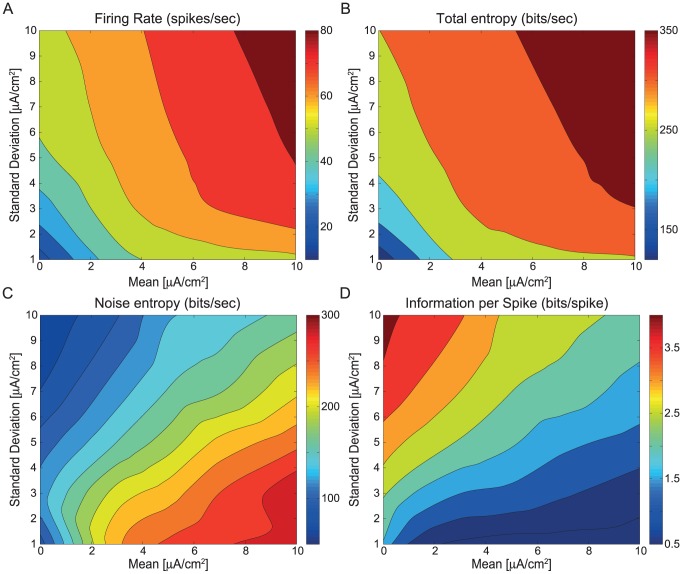
The effect of stimulus statistics upon the rate, timing and precision of action potentials. A. Firing rates (spikes s^−1^) of the spiking neuron model evoked by white noise current stimuli with different means and standard deviations. The current stimuli used in A–D were identical to those in Figure 3A. B. Total entropy (bits s^−1^), C. Noise entropy (bits s^−1^), and D. Information rate per spike (bits spike^−1^) of the spiking neuron model.

Information coding in spiking neurons is dependent upon the rate and timing of the action potentials with which it samples the input stimulus [27]. We calculated the firing rate of the spiking neuron model in response to the same set of band-limited white noise current stimuli used previously to calculate information rates (see Methods) (Figure 3A). Increasing the stimulus mean or standard deviation increased the firing rate; low mean, high standard deviation or high mean, low standard deviation stimuli produced approximately 57 spikes/s whereas high mean, high standard deviation stimuli generated the highest spike rates of approximately 86 spikes/s (Figure 3A). Because these firing rates are lower than the maximum firing rates that the spiking neuron model can achieve, the information rates are not limited by the absolute refractory period.

The total entropy of a spike train reflects its total variability over time [26]. The highest total entropy occurred with high mean, high standard deviation stimuli that produced the highest spike rates, conversely, the lowest total entropy occurred with low mean, low standard deviation stimuli that produced the lowest spike rates (Figure 3B). However, noise prevents neurons from achieving the maximal information rates, as bounded by the total entropy [26]. We quantified the differences in action potential reliability by calculating the noise entropy among spike trains generated by many repetitions of an identical current stimulus (Figure 3C). Increasing the stimulus standard deviation increased the number of transients in the stimulus that cross the voltage threshold at high velocity. Consequently, high standard deviation stimuli generated spike trains that were both precise and reliable among trials with low noise entropy (Figure 3C; Figure S1A) [28]. Conversely, as the mean increased the variance in the interspike interval influenced spike timing, reducing the reliability of the spike trains and increasing the noise entropy (Figure 3C; Figure S1B).

The total and noise entropy together determine the information rate of the spiking neuron model for a particular input stimulus (Figure 2A). Low mean, high standard deviation stimuli generated spike trains that have only intermediate firing rates and total entropies but have the highest information rates due to their low noise entropy. High mean, high standard deviation stimuli generated spike trains with lower information rates despite their higher firing rates because noise entropy is higher. Consequently, the information per spike was highest (4 bits/spike) with low mean, high standard deviation stimuli that produced the highest information rates (235 bits/s) with only moderate firing rates (57 Hz), and lowest (0.2 bits/spike) with high mean, low standard deviation stimuli that produced the lowest information rates (10.2 bits/s), also with moderate firing rates (58 Hz) (Figure 3D).

### Channel noise and spiking

To determine the effect of noise generated by the voltage-gated Na^+^ and K^+^ channels on the information rates of the spiking neuron model, we replaced either the stochastic Na^+^ or K^+^ channels with deterministic channels thereby eliminating this component of the channel noise. In comparison to the stochastic model, the deterministic Na^+^ channel model generated more reliable spike trains for a given stimulus (Figure S1A,S2A). Similarly, replacing the stochastic K^+^ channels in the spiking neuron model with deterministic channels also generated more reliable spike trains for a given stimulus in comparison to the original spiking neuron model (Figure S1A,S2B).

**Figure 4:**
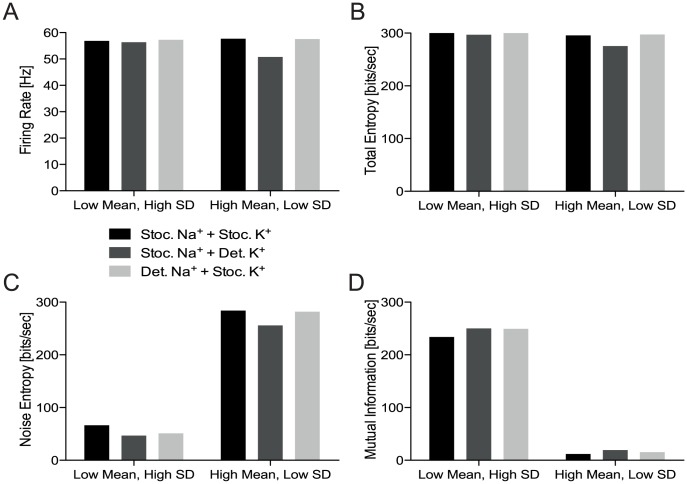
The information rates of the spiking neuron model are robust to voltage-gated ion channel noise. A. The firing rates of the spiking neuron model (stochastic voltage-gated Na^+^ and K^+^ channels), a modified model with stochastic voltage-gated Na^+^ and deterministic voltage-gated K^+^ channels and a modified model with deterministic voltage-gated Na^+^ and stochastic voltage-gated K^+^ channels evoked by low mean, high standard deviation or high mean, low standard deviation input stimuli. B. The total entropy, C. The noise entropy, and D. The mutual information rates of the same models shown in A evoked by the same stimuli.

We quantified differences in the reliability between the original stochastic spiking neuron model, the modified model with deterministic Na^+^/stochastic K^+^ channels, and the modified model with stochastic Na^+^/deterministic K^+^ channels. We compared the total entropy, noise entropy, information rate and information per spike for spike trains generated by low mean, high standard deviation stimuli or high mean, low standard deviation stimuli (Figure 4). All three models produced 50–57 spikes/s in response to the stimuli (Figure 4A). In comparison to the original spiking neuron model with stochastic Na^+^ and K^+^ channels, the total entropy of the deterministic K^+^ channel model was lower by 1–7%, whereas the total entropy of the deterministic Na^+^ model was almost identical (Figure 4B). The deterministic K^+^ channel model also had the lowest noise entropy, making the APs more reliable (Figure 4C). Both models with deterministic ion channels had higher information rates than the original model because of their lower noise entropy, but the difference was just 7%, irrespective of the stimulus statistics (Figure 4D). This suggests that channel noise has relatively little impact on the information rate of the 100 µm^2^ single compartments we modelled. Thus, in addition to channel noise and dimensionality reduction, there must be other sources of information loss.

### Information encoded in the generator potential

**Figure 5:**
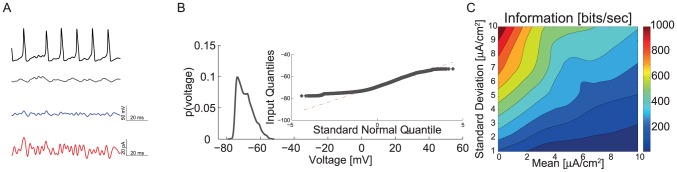
The information encoded in the pseudo-generator potentials of the spiking neuron model. A. Action potentials (top black trace) evoked by white noise current stimuli (bottom red trace). Upper grey trace: The same voltage response with the action potentials removed and replaced with a linear interpolation of the voltage. This is the pseudo-generator potential, which is an approximation of the generator potential. Lower blue trace: A voltage response of the graded neuron model to the current stimulus shown in the bottom trace. B. The PDF of the pseudo-generator voltage response. (Inset) A QQ plot showing departures from a Gaussian distribution (dotted red-line) for the time-series shown in A (upper grey trace). C. Information rates (bits s^−1^) of pseudo-generator potentials evoked by white noise current stimuli with different means and standard deviations. The stimuli are identical to those in Figures 2 and 3.

The information in the spike train comes from the generator potential (Figure 5A). However, the generator potential is not equivalent to the voltage signals produced by the graded potential model, which lacks voltage-gated Na^+^ channels. We constructed an approximation of the generator potential, the pseudo-generator potential, by removing the action potentials from spike trains and replacing them with a 6 ms linear interpolation of the membrane potential, corresponding to the maximum action potential width (Figure 5A). The pseudo-generator potential probability density function is distorted in comparison to the graded potential being narrower with a more pronounced peak because voltage excursions beyond threshold are truncated, the action potential being replaced with an interpolated response (Figure 5A,B). For a particular stimulus the information rate of the pseudo-generator potential was intermediate between that of the spike trains and that of the graded potential model (Figure 5C). The information rates of the pseudo-generator potential were highest (1094 bits/s) with low mean, high standard deviation stimuli, 860 bits/s (366%) higher than that of the corresponding spike trains but 1146 bits/s (51%) lower than that of the corresponding graded potential (Figure 5C). The information rates of the pseudo-generator potential were lowest (188 bits/s) with low mean, low standard deviation stimuli. This lowest value was 158 bits/s (531%) higher than that of the corresponding spike trains, but 352 bits/s (65%) lower than that of the corresponding graded potential (Figure 5C).

What reduces the information rate of the pseudo-generator potential relative to the graded potential? We identify three processes: the duration of the action potential and associated refractory period, and two effects caused by the presence of voltage-gated Na^+^ channels, noise and non-linearity. We will assess each of these processes, in turn.

### Information loss during the action potential

**Figure 6:**
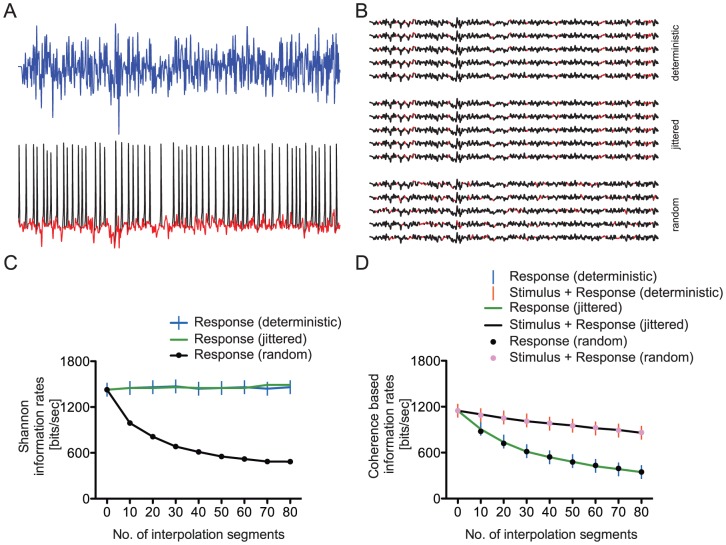
The action potential ‘footprint’ reduces the information encoded in a graded voltage response. A. White noise current (blue) elicits a train of action potentials in the spiking neuron model (black). The same voltage response with the action potentials removed and replaced with a linear interpolation of the voltage (red). B. Sections of the graded voltage response were replaced with a linear interpolation to mimic the ‘footprint’ each action potentials creates when any information contained in the graded response is obscured. The graded responses are shown in black and the interpolated sections in red. The 3 replacement regimes deterministic (upper), jittered (middle) and random (lower) mimicked spiking statistics with different current stimuli (see main text for details). C. The random insertion of 6 ms sections of linear interpolation into graded voltage responses reduces the Shannon information rate. The drop in information rate is greater with more interpolations. Insertion of interpolations in the same (deterministic) or nearly the same (jittered) positions does not affect the Shannon information rate. D. The random insertion of 6 ms sections of linear interpolation into graded voltage responses reduces the coherence-based information rate. The drop in information rate is greater with more interpolations. The insertion of interpolations in the same (deterministic) or nearly the same (jittered) positions have the same effect upon the coherence-based information rate as the random insertion. Insertion of interpolations into the same positions within the stimulus as well as the response reduces the effect upon the coherence-based information rate.

The action potential and accompanying refractory period creates a ‘footprint’ on the generator potential during which information is lost (Figure 6A). To assess the impact of this ‘footprint’ on the information rate, we stimulated the graded model with a white noise stimulus (Figure 1A,B) to generate a set of graded responses from which we could estimate the signal, noise and information rate. These graded responses produced a high information rate (1427 bits/s). We then inserted 6 ms long sections of linear interpolation spaced at least 10 ms apart into the individual graded responses to mimic action potential footprints (Figure 6B). We added between 10 and 80 linear interpolations per second into each response to represent the spike footprints at different firing rates and re-calculated the Shannon information rate (Figure 6B) [25]. Interpolations were added at exactly the same positions in all responses, termed *deterministic* interpolation (Figure 6B), to represent the footprints of noise-free spikes and give an upper bound on signal entropy. The placement of the interpolations was then jittered by up to 4 ms (Figure 6B), termed *jittered* interpolation, to represent reliable spike trains with low noise entropy. Finally, interpolations were placed randomly in each response (Figure 6B), termed *random* interpolation, to resemble unreliable spike trains with high noise entropy.

The Shannon information rate [25] was unaffected by the *deterministic* or *jittered* interpolation, irrespective of the number of interpolations inserted (Figure 6C) because it depends only upon the signal-to-noise ratio (SNR) and the response bandwidth [25]. Thus, inserting increasing numbers of interpolations, even when jittered, does not affect the Shannon information rate because these interpolations are inserted in identical (*deterministic*) or similar (*jittered*) positions, leaving the regions between the interpolations unaffected. Conversely, increasing the number of *random* interpolations reduced the Shannon information rate from 1427 to 485 bits/s (Figure 6C) because these interpolations add noise to the responses, thereby reducing the SNR.

In addition to the Shannon information rate [25], we calculated coherence-based information rates to determine the effect of the footprint on information loss from the stimulus (see Methods). The coherence-based estimate of the information rate is a measure of linear dependence between the stimulus and the response, and describes different forms of signal corruption including non-linear distortion [29]. The coherence-based information rate decreased as the number of interpolations inserted increased for all three types of interpolation, *deterministic*, *jittered* and *random* (Figure 6D). The coherence-based information rate dropped from 1148 bits/s with no interpolations to 346 bits/s with 80 interpolations.

Although we inserted linear interpolations into the voltage responses, there is still a fluctuation at the corresponding position in the current stimulus. The mismatch between the interpolations and the stimulus may reduce the coherence-based information rate by inflating the non-linearity. To determine whether this is the case, we added linear interpolations at exactly the same positions to both the stimulus and the response, and recalculated the coherence-based information rate (Figure 6D). This difference between the coherence-based information rates calculated with or without interpolations added to the stimulus as well as the response is the information lost due to the action potential footprint. For the same number of interpolations, all three types of interpolation, *deterministic*, *jittered* and *random*, had higher information rates (between 177 and 516 bits/s) with interpolations added to the stimulus than without (Figure 6D). These coherence-based information rates were dependent upon the number of interpolations inserted. For example, inserting 10 interpolations reduced the information rate from 1148 bits/s to 1090 bits/s but inserting 80 interpolations reduced the information rate to 860 bits/s. Thus, the coherence-based method demonstrates that the action potential footprint blanks out information about the stimulus. This loss of information increases with spike rate from 5.3% at 10 Hz to 33.5% at 80 Hz.

### Sub-threshold noise

**Figure 7:**
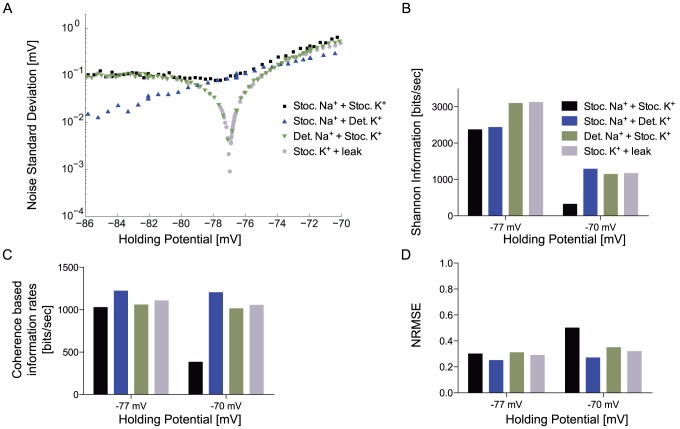
The effects of channel noise upon sub-threshold and graded voltage signals. A. The standard deviation of the voltage of the spiking neuron model (stochastic voltage-gated Na^+^ and K^+^ channels), a modified model with stochastic voltage-gated Na^+^ and deterministic voltage-gated K^+^ channels, a modified model with deterministic voltage-gated Na^+^ and stochastic voltage-gated K^+^ channels, and the graded neuron model (stochastic voltage-gated K^+^ channels) over a 16 mV range of holding potentials. B. Shannon information rates of all four models shown in A evoked by low mean, high standard deviation current stimuli at sub-threshold holding potentials. C. Coherence-based information rates of all four models shown in A evoked by low mean, high standard deviation current stimuli at sub-threshold holding potentials. D. Normalized mean square error (nRMSE) information rates of all four models shown in A evoked by low mean, high standard deviation current stimuli at sub-threshold holding potentials.

Channel noise affects sub-threshold potentials as well as spike timing and reliability [30]. We measured the standard deviation of the voltage noise at sub-threshold membrane potentials for the spiking neuron model, the deterministic Na^+^/stochastic K^+^ channel model, the stochastic Na^+^/deterministic K^+^ channel model and the graded neuron model (Figure 7A). In the absence of an input stimulus, the voltage noise was generated entirely by the spontaneous opening and closing of the voltage-gated ion channels. The noise standard deviation of all the models was highest at the most depolarised potentials and dropped as the membrane potential was hyperpolarised towards the reversal potential of the K^+^ ions (Figure 7A). Between −74 to −70 mV the voltage noise standard deviation was highest for the spiking neuron model and lowest for the stochastic Na^+^/deterministic K^+^ channel model.

The voltage noise of the deterministic Na^+^/stochastic K^+^ was close to that of the spiking neuron model (Figure 7A). However, near the K^+^ reversal potential of −77 mV the voltage noise of all three models containing stochastic K^+^ channels dropped as the driving force on K^+^ ions approached zero. The drop was less pronounced in the spiking neuron model because stochastic Na^+^ channels continued to produce noise. Below the K^+^ reversal potential, the voltage noise of all three models containing stochastic K^+^ channels increased (Figure 7A), with the driving force on K^+^ ions.

The voltage noise of the deterministic K^+^ channel model dropped as the membrane potential was hyperpolarised, even below K^+^ reversal potential, because the probability of spontaneous Na^+^ channel opening, the only source of channel noise, drops at hyperpolarised potentials. Indeed, the deterministic K^+^ channel model had the lowest voltage noise at holding potentials more depolarised than ∼−74 mV and more hyperpolarised than ∼−80 mV (Figure 7A). Thus, although the noise generated by the spontaneous opening of both Na^+^ and K^+^ channels contributes to the voltage noise of the spiking neuron model, the K^+^ channel noise apparently makes the greater contribution at potentials between −74 to −70 mV. Note that the voltage noise standard deviation with both channel types together is less than the sum of the standard deviations of the individual channel types because their variances add.

We assessed the impact of the sub-threshold voltage noise on the Shannon information rate by stimulating each model with a white noise current with a zero mean and low standard deviation (μ = 0, σ = 1, τ_c_ = 3.3 ms). An additional tonic current was injected and adjusted to hold the mean membrane potential at either −77 or −70 mV. This tonic current prevented the models containing voltage-gated Na^+^ channels from reaching threshold, permitting a direct comparison of the effects of stochastic and deterministic channel combinations upon sub-threshold information coding.

We calculated the Shannon information rate [25] of each model at the two mean potentials, −77 and −70 mV (Figure 7B). The highest information rates of all the models occurred at the more hyperpolarised potential because the voltage noise was lower. Due to a distinct drop in voltage noise near the K^+^ reversal potential, the deterministic Na^+^/stochastic K^+^ channel model and the graded neuron model, attain the highest information rates of 3123 bits/s at −77 mV. These information rates were ∼30% greater than those of the sub-threshold spiking neuron model and the stochastic Na^+^/deterministic K^+^ channel model, which are lower because of voltage-gated Na^+^ channel noise. At −70 mV the increased voltage noise in all the models reduces their information rates (Figure 7B). The information rate of the sub-threshold spiking neuron model dropped 86% to 321 bits/s. The sub-threshold information rates of both models with stochastic K^+^ channels dropped 63% to 1142–1168 bits/s, whilst the stochastic Na^+^/deterministic K^+^ channel model has the lowest voltage noise and, consequently, the highest sub-threshold information rate of 1288 bits/s. The drop in the information rates of all the models at the more depolarised holding potential shows the substantial effect of channel noise upon the sub-threshold and graded potentials. The combination of both stochastic Na^+^ and stochastic K^+^ ion channels in the spiking neuron model reduce the information content of the sub-threshold potential relative to the graded neuron model by 24% at −77 mV to 73% at −70 mV.

### Sub-threshold non-linearity

Voltage-gated ion channels introduce non-linearities [31], [32] that could reduce the information content of the generator potential by distorting the voltage signal. We assessed the sub-threshold effect of non-linearity on each of the models, at −77 mV and −70 mV, using the coherence-based information rates we previously calculated to assess the impact of the action potential footprint (Figure 6D). Higher coherence-based information rates indicate better reconstruction of the original stimulus, based solely on linear decoding principles [29]. In the spiking neuron model the coherence-based information rates dropped by more than 63% as the holding potential becomes more depolarised i.e., from 1027 bits/s at −77 mV to 382 bits/s at −70 mV (Figure 7C). This fall indicates a decline in the quality of linear reconstruction. By comparison, the stochastic Na^+^/deterministic K^+^ model was the least affected by depolarisation, the coherence-based information rates dropping by just 1.5%. For the model with deterministic Na^+^/stochastic K^+^ and the model with only stochastic K^+^ channels, the coherence-based information rates drop ∼4.2–4.8% at the more depolarised potential (Figure 7C). Increasing the holding potential to −68 mV causes all three models containing voltage-gated Na^+^ channels to produce spikes, making them increasingly non-linear (data not shown).

In addition to coherence-based information rates, we used the normalised root mean squared error (nRMSE) between the original stimulus and the reconstructed stimulus to assess the effect of non-linearity. An nRMSE value that tends towards zero represents perfect reconstruction [29]. The nRMSE increased as the membrane potential increased indicating a drop in the quality of reconstruction (Figure 7D); the increase in nRMSE was largest for the sub-threshold spiking model (67%) but the nRMSE of the three other models also increased by 8–13%. This decline in reconstruction quality is due to an increase in the open channel probability with depolarization. For the models containing voltage-gated Na^+^ channels, the voltage threshold for eliciting an action potential is close to −68 mV. At −70 mV the increase in the numbers of open voltage-gated Na^+^ channels increases positive-feedback and, consequently, the magnitude of the non-linearity. A fluctuating input stimulus superimposed upon the holding current also reduces the distance from the voltage threshold, though the effect of this on reconstruction will depend on the magnitude and polarity of the fluctuations.

### Linear decoding accuracy in the suprathreshold regime

Using linear systems analysis (see Methods), we assessed how much of the input (current) can be predicted from the response (voltage) by reconstructing the input stimulus current. We find that when the graded voltage response was used for the reconstruction based on linear decoding the predicted input stimuli were most coherent, with the lowest nRMSE (Figure S3C,D) and the highest coherence-based information rates (Figure S3C,E). The reconstruction accuracy (nRMSE and coherence based information) of the pseudo-generator potentials was lower than that of the graded potentials (Figure S3B,D,E). The highest nRMSE and, consequently, the lowest coherence-based information rate was obtained from reconstructions based on action potentials (Figure S3A,D,E), although these were only marginally worse than reconstructions based on pseudo-generator potentials (Figure S3). Thus, voltage-gated Na^+^ channels distort both the subthreshold (pseudo-generator) and suprathreshold responses so that the incoming stimulus current cannot be accurately reconstructed using just a linear decoder.

### Extrinsic noise

Neuronal information rates are constrained by extrinsic noise in the input stimuli, as well as by intrinsic noise generated by ion channels [33], [34]. To investigate this constraint, we added broadband Gaussian noise to the white noise input stimulus. This enabled us to quantify and compare the effect of extrinsic noise upon the information rates of the spiking model, the pseudo-generator potentials from the spiking model and the graded model. In our simulations, although the presence of the extrinsic noise source facilitates a marginal increase in precision of the APs for inputs with low standard deviations, it does not alter the variability of the APs, consequently noise-aided enhancement of mutual information is absent (*cf.* McDonnell et al. [35]).

The amount of extrinsic noise was altered to produce an input stimulus with either a low or a high SNR input stimulus (Equations 2 and 3; SNR = 2 or 20). The SNR is defined as the ratio of the signal power to the noise power. In our simulations, we decreased the SNR by increasing the noise power (see Methods; Equation 3). For the spiking model, increasing the input noise produces a relatively small increase in total entropy (∼5%, SNR = 2; ∼2%, SNR = 20) (Figure S4A) but a relatively large increase in noise entropy (∼180%, SNR = 2; ∼50%, SNR = 20) (Figure S4B), and this produces a significant drop in the mutual information (∼40%, SNR = 2; ∼10%, SNR = 20) (Figure S4C,S5A).

The information rates of the pseudo-generator potentials also decrease with increased extrinsic noise (Figure S5B). The loss in relation to the noise-less stimulus is greater in the pseudo-generator potentials (∼69%, SNR = 2; ∼29%, SNR = 20) with higher standard deviation input signals. A 10-fold increase in the input SNR caused a 133% increase in information rate, from 335 bits/sec (SNR = 2) to 780 bits/sec (SNR = 20), compared to 1094 bit/sec in the absence of extrinsic noise. Likewise, the information rates of the graded model were reduced by up to 73% for low SNR input signals (SNR = 2) and by up to 36% for high SNR input signals (SNR = 20) (Figure S4C), the higher quality input signal (SNR = 20) causing the information rate to increase from 595 to 1422 bits/sec. Thus, the information rates of the spiking model were the least affected by the extrinsic noise whilst those of the graded model were the most affected (Figure S5A–C).

### Energy consumption

**Figure 8:**
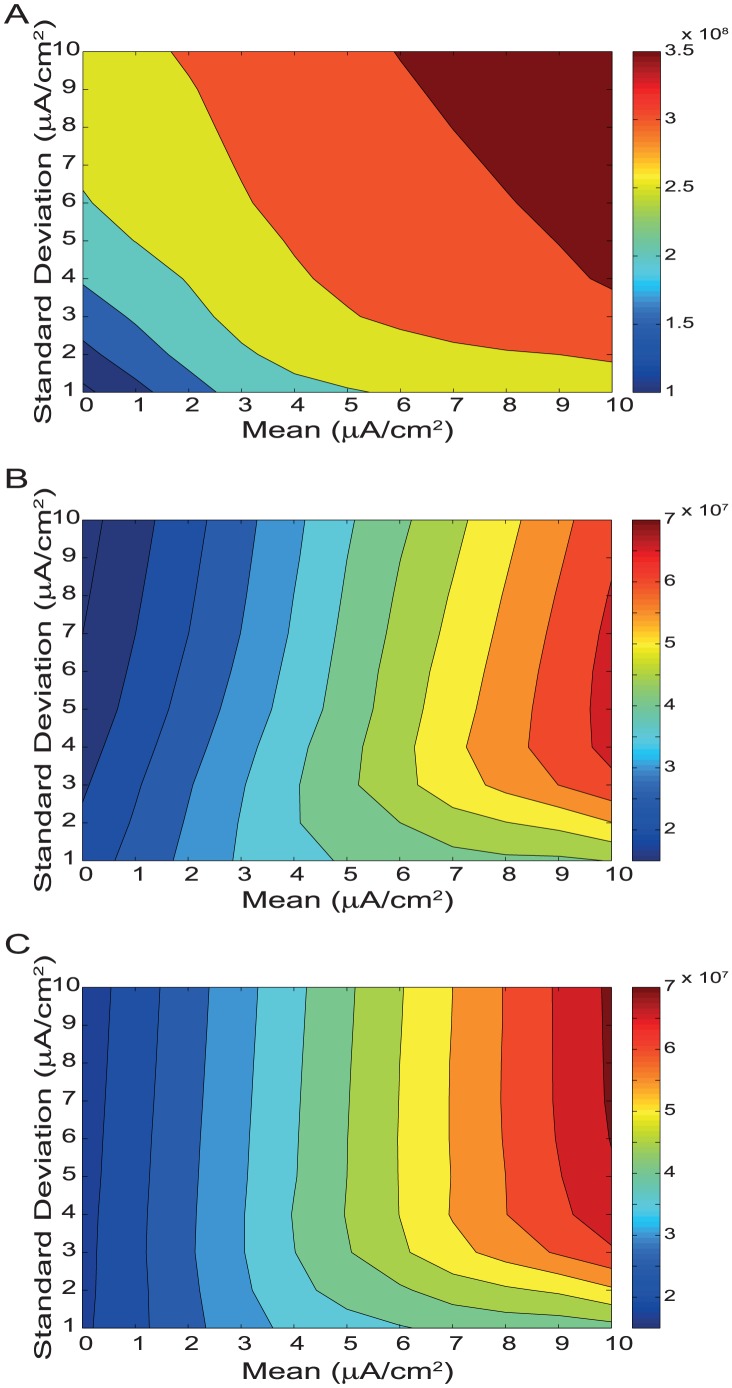
The energy consumption of spike trains, pseudo-generator potentials and graded potentials. A. Energy consumption (ATP molecules s^−1^) of the spiking neuron model, B. the pseudo-generator potentials, and C. the graded potential model evoked by white noise current stimuli with different means and standard deviations.

The energy consumption of each model was determined from the K^+^ ion fluxes across the membrane needed to generate the voltage signals, as the number of ATP molecules hydrolyzed by the Na^+^/K^+^ pump [12]. This pump maintains the ionic concentration gradients that generate electrical responses and operates stoichiometrically, pumping back 2 K^+^ ions for every ATP molecule that it consumes [36]. The energy consumption of the spiking neuron model is strongly correlated with its firing rate (Figure 8A) because the energy consumption of an action potential is high compared to the consumption between action potentials. Higher standard deviation stimuli evoke larger membrane potential fluctuations, eliciting more action potentials and, therefore, consuming more energy. Consequently, the high mean, high standard deviation stimuli that evoked the highest firing rates also incurred the highest energy consumption, 3.9*10^8^ ATP molecules/s (Figure 8A). Low mean stimuli with high standard deviations consume 3.1 times more energy than stimuli with low standard deviations but for high mean stimuli it is just 1.4 times more (Figure 8A). This is because the standard deviation of signal fluctuations has less of an effect upon the average firing rate with high mean input stimuli.

Pseudo-generator membrane potentials consume less energy than the spiking neuron model. Indeed the maximum energy consumption of the pseudo-generator potentials is 6.4*10^7^ ATP molecules/s, almost an order of magnitude less than the spiking neuron model (Figure 8B). Like the spiking model, when the pseudo-generator potential model is driven with a high mean stimulus, increasing the stimulus standard deviation increases energy consumption. But, unlike the spiking model, when the stimulus mean is low, increasing its standard deviation reduces energy consumption. Low mean, high standard deviation stimuli consume less energy because they hyperpolarise the membrane potential by 10 mV or more below the resting potential, and this reduces the number of open K^+^ channels (Figure S6A,B). Conversely, with high mean stimuli the maximum peak-to-peak voltage of the compartment is approximately the same, irrespective of the standard deviation (Figure S6A,B). The greater energy consumption of the high standard deviation is due to the 1.6-fold greater numbers of open K^+^ channels, which cause a doubling of the mean K^+^ current at equivalent membrane potentials, thereby inflating the energy consumption.

The energy consumption of the graded model showed the same trends as the pseudo-generator potentials (Figure 8C). Again, less energy is consumed in response to low mean high standard deviation stimuli than to low standard deviation stimuli, due to an 85% decrease in the number of open K^+^ channels (Figure S7A,B). In contrast, at high means, high standard deviation stimuli consumed 64% more energy than low standard deviation stimuli (Figure 8C) because high input standard deviations open greater numbers of K^+^ channels (Figure S7A,B).

### Energy efficiency

**Figure 9:**
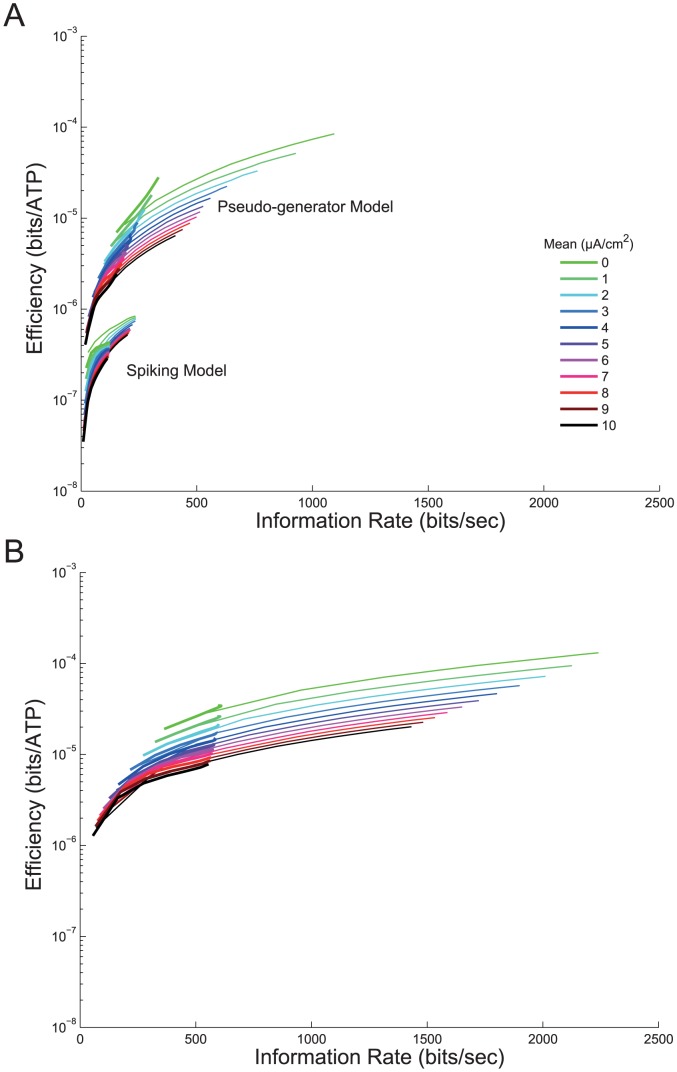
The energy efficiency of information encoding in spike trains, pseudo-generator potentials and graded potentials. A. Energy efficiency of information processing (bits ATP molecule^−1^) with (thick lines; SNR = 2) and without (thin lines) extrinsic noise in the spiking neuron model and the model containing the pseudo-generator potentials, and B. the graded potential model evoked by white noise current stimuli with different means and standard deviations.

We calculated the energy efficiency of information coding by dividing the information rates of the spiking neuron model, the pseudo-generator potentials and the graded neuron model by their corresponding energy consumptions. The energy efficiency of the spiking neuron model was highest (8.4*10^−7^ bits/ATP molecule) for low mean, high standard deviation stimuli and lowest (3.8*10^−8^ bits/ATP molecule) for high mean, low standard deviation stimuli (Figure 9A). This 22-fold difference in energy efficiency was accompanied by a 23-fold difference in information rate. Thus the coding of low mean, high standard deviation stimuli was most efficient because these stimuli generated the highest information rates with firing rates, and therefore energy costs, similar to high mean, low standard deviation stimuli (Figure 9A). In other words, energy efficiency rises with information per spike.

Indeed, in all models, spiking, pseudo-generator potential, and graded, increasing input stimulus mean reduced energy efficiency because it increased the mean level of response without introducing more information (Figure 9A,B). As expected, the energy efficiency of all three models improved when the information rate increased in response to an increase in stimulus standard deviation at a given stimulus mean (Figure 9A,B). For example at low means, the spiking model's information rate increased by 689% with a concomitant increase in efficiency of 151%. For the pseudo-generator potentials information increased by 482%, and efficiency increased by 889% and in the graded neuron efficiency increased by 363% and information increased by 315%.

Both pseudo-generator (8.0*10^−5^ bits/ATP) and graded potential (1.3*10^−4^ bits/ATP) models were 95–156 times as energy efficient as the spiking model (8.4*10^−7^ bits/ATP), when all models were compared with low mean, high standard deviation inputs. At higher information rates the energy efficiency of both the pseudo-generator and graded potentials improved substantially (Figure 9B). However, the graded potentials achieved higher information rates than the pseudo-generator potentials and in this regime they were as much as 1.6 times more energy efficient at 1.3*10^−4^ bits/ATP molecule.

The addition of extrinsic noise did not affect this general pattern of relationships between input stimuli, information rate and energy efficiency in the three models. However, by reducing the information rates of all three models the extrinsic noise reduced the energy efficiency for any given input stimulus (Figure 9A,B). For example, adding noise to the inputs reduced the efficiencies of the pseudo-generator potentials by 71% for a low quality input (SNR = 2) and by 26% for a high quality input (SNR = 20). Similarly, the efficiency of the graded potential model dropped by 74% at low SNR and by 36% for high input SNR. Given that extrinsic noise only marginally altered the energy consumption, it decreases efficiency by decreasing the amount of information that can be coded.

## Discussion

Analogue voltage signals in non-spiking neurons and generator potentials in spiking neurons typically have higher information rates than spike trains [5]–[9]. This information loss is a consequence of a change in coding strategy; non-spiking neurons and generator potentials encode information as a continuous analogue voltage signal whereas spiking neurons use discrete pulses of finite precision and width, limiting the number of states that can be coded within a given time period. However, there are also biophysical causes of this information loss, and these were the focus of our study. Spiking neurons can be lossless encoders of band-limited inputs if their spike rates exceed the Nyquist limit [37], both at the level of a single neuron or across a population of neurons [38], [39]. But below this limit information loss occurs and is affected by the factors we have examined.

Our simulations show that voltage-gated Na^+^ channels, which are necessary for action potential generation, are the primary biophysical cause of information loss in sub-threshold potentials because they increase intrinsic noise and introduce non-linearities. Indeed, this information loss in sub-threshold potentials is greater than the information loss in spike generation attributable to voltage-gated Na^+^ channels. Further information loss in the sub-threshold potential occurs because each action potential obscures the generator potential, reducing its information content. This suggests that the biophysical factors we identify have their major impact upon sub-threshold information processing. Comparing the energy efficiencies of our models, spike trains consume an order of magnitude more energy than graded or pseudo-generator potentials for a given stimulus. This result emphasizes the two-fold penalty of action potentials on coding efficiency; lower information rates and higher energy costs. Graded and generator potentials consume similar amounts of energy, the primary determinant of which is the input mean, but due to their lower information rates generator potentials are less energy efficient than graded potentials.

Our models contained voltage-gated ion channels with the same biophysical properties as those found in the squid giant axon because well-established kinetic models exist for them [40], [41]. Different channel kinetics will alter channel noise [42], affect the shape of the action potential [11] and alter the information rates of a spiking model [43]. However the main effects of channel noise in our models are on the graded and generator potentials. Previous modeling studies have used squid voltage-gated Na^+^ channels to show that they increase sub-threshold noise [30], but did not quantify their effect on sub-threshold information rates. We find that the noise from voltage-gated Na^+^ channels and voltage-gated K^+^ channel noise substantially decreases the information rate of the generator potential. This finding suggests that the high densities of voltage-gated Na^+^ channels at the spike initiation zone [44], as well as voltage-gated Na^+^ channels and Ca^2+^ channels in dendrites and dendritic spines [45], [46] could also reduce the information rate of sub-threshold signals, and this could have a deleterious effect on information processing.

Our models suggest that action potential duration (including the absolute refractory period) is an important source of information loss, imposing a lower limit on the interspike interval and preventing the spike initiation zone from integrating new information for a brief period. *In vivo* many neurons have considerably higher spike rates than our models, which had moderate spike rates below approximately 90 Hz. At these high spike rates, substantial portions of the information would be lost from the generator potential, promoting narrower action potentials and sparse codes that require relatively few action potentials [47]. However, many neurons use signals that are considerably longer than typical action potentials such as bursts and plateau potentials [48] that obscure far more of the generator potential and incur a greater information loss. This emphasizes the importance of these long-duration signals as indicators of high salience signals.

The non-linearity of all the models incorporating voltage-gated Na^+^ channels increases with the sub-threshold depolarization because the positive feedback generated by the Na^+^ channels increases as the threshold approaches. Thus, at sub-threshold levels the Na^+^ channels distort the voltage waveform. This distortion could reduce the information content of the sub-threshold potentials, though this depends upon whether the transformation of any synaptic metric (current, conductance, etc.) into the voltage waveform is linear in the sub-threshold regime. Linear as well as non-linear mapping may occur between the synaptic input and the resultant voltage waveform [20], [49]. Voltage-gated Na^+^ channels may constitute one such non-linearity, distorting the synaptic input [50]. In such cases, although a linear decoder cannot fully represent and recover the input information, a decoder relying on higher order features of the membrane potential may prevent any information loss (also see [39]).

Our use of current rather than conductance as the input stimulus ignores the energy cost associated with conductance inputs, which will reduce the energy efficiency of information coding of all the models. Conductance inputs close to the spike initiation zone will also alter the membrane time constant and affect action potential initiation [51], [52]. Consequently, conductance inputs will affect the bandwidth and temporal precision of all the models and the maximum spike rate of the spiking neuron model [53]. The synaptic channels needed to implement the conductance changes will also contribute noise to the models [54], reducing their information rates. By incorporating extrinsic noise, however, we have shown that the relationships we have found will remain qualitatively similar.

The squid giant axon action potentials that we modeled consume substantially more energy than other vertebrate and invertebrate action potentials [11], [14]–[16], inflating the energy consumption of the spiking neuron model and reducing its efficiency. Nevertheless, the efficiency drop that occurs when generator potentials are converted to action potentials is substantial and will remain, albeit with a smaller difference. The topological class of model (e.g. Type I or Type II) may also influence energy consumption through the dynamics and time course of the ionic and synaptic currents determining the threshold manifold [55]. Indeed, minimizing metabolic consumption in single compartment models [55] leads to the leak and the inward currents competing with each other even before reaching the spiking threshold, via a Hopf bifurcation (Type II). This causes an increase in energy consumption forcing the optimal action potentials to steer away from such bifurcations; gradient descent on metabolic consumption leads to saddle-node bifurcations as in Type I cortical neurons (unpublished observation – BS, personal communication – Martin Stemmler, [55]). The energy consumption of the graded potential neurons will also be affected by changes in the biophysical properties of voltage-gated ion channels, though this is unlikely to substantially affect the relationship between the input stimuli and the energy consumption.

Our models systematically explored combinations of the mean and standard deviation of a Gaussian input. Those spike trains with the lowest information rates and bits per spike were evoked by low standard deviation stimuli, whereas high standard deviation stimuli evoked consistently higher information rates for a given mean stimulus. Consequently, across all our models there was no systematic relationship between the mean spike rate and the information rate, total entropy, noise entropy or coding efficiency (bits per spike). Indeed, the highest and lowest information rates and coding efficiencies were found at similar spike rates. However, these findings are specific to the type of stimuli we used, a randomly varying input signal superimposed on an offset. It is more usual to find that the information rate increases with spike rate whilst the coding efficiency declines because the entropy per spike falls [56], [57]. Non-Gaussian naturalistic stimuli vary more widely than do Gaussians. These larger excursions make the voltage response more nonlinear and engage adaptation mechanisms that, if they affect the signal and noise differently, can change the information rates of both graded potentials and the spike trains they generate [58]. Although there are methods that could allow us to compare the coding and metabolic efficiency of analogue and spiking responses to natural stimuli [6], each modality has its own statistics. Even within a modality different classes of neuron have distinctive firing patterns because they select different components of the input (e.g. retinal ganglion cells [57]). Faced with many particular cases, we chose to start with a general stimulus that identifies factors, such as input signal to noise, that are widely applicable.

As a case in point, in many neurons the mean and standard deviation of the input stimuli and the extrinsic noise are often correlated [59]–[61]. For example, extrinsic noise in synaptic inputs is often correlated with their number and strength, and hence signal amplitude [62]–[64]. Thus, the stimulus space investigated with our models exposes relationships between energy efficiency and information rate that are broadly applicable to a number of different types of neuron. In particular, our models demonstrate that the energy efficiency of spiking neurons can be improved by reducing the mean input and increasing the standard deviation of the signal. Graded neurons achieve this by using predictive coding to eliminate the mean and amplify the remaining signal to fill their output range [65], [66] and these procedures increase both their coding efficiency and their energy efficiency [3]. Our findings demonstrate that spiking neurons can do likewise.

Taken together, our analyses show that the biophysical mechanisms involved in action potential generation contribute significantly to the information loss that accompanies the conversion of a graded input to a spike train. Although we cannot directly relate the proportions of information loss to specific mechanisms, it seems likely that the action potential ‘footprint’ and sub-threshold voltage-gated Na^+^ channel noise are the major sources of information loss. Viewed as a cost-benefit trade-off, action potentials incur penalties (information loss and energy cost) that are, presumably, balanced against being able to transmit information over considerable distances and preventing noise accumulation during successive processing stages. Reducing the distances over which information is transmitted in the nervous system may favor less conversion of graded signals into spike trains [67]. However, problems associated with accumulating noise during successive processing stages [4] may remain severe. Thus, even in some highly miniaturized nervous systems, neurons with action potentials are likely to be necessary [67].

In conclusion, our modeling of single compartment neurons confirms that a critical step in neural coding, the conversion of an analogue sub-threshold signal to a series of discrete “digital” pulses, is accompanied by substantial information loss. We show that voltage-gated Na^+^ channels, critical components for the conversion of analogue to digital, reduce the information in sub-threshold analogue signals substantially, and that this loss is compounded by interference from action potentials. Thus, the first step in a hybrid processing strategy to increase efficiency [4], [68], the analogue processing of inputs, is compromised by mechanism used for the second step, the conversion of analogue to digital, and this calls for strategic placement of the spike initiation zone [69]. Some neurons appear to mitigate a small fraction of the loss of information that accompanies the conversion of analogue to digital by transmitting both analogue and digital [70]–[72]. Information may be encoded in the height and width of action potentials [71]–[74] suggesting that spiking neurons may transmit more information than is calculated by treating them as digital pulses. Even in these cases, however, the ‘footprint’ of the action potentials and sub-threshold voltage-gated Na^+^ channel noise are still likely to cause substantial information loss.

## Methods

### Single compartment model

We used a single compartment stochastic Hodgkin-Huxley model of the squid giant axon for our simulations [40], [41]. The model supporting spiking contained two voltage-gated ion channels, transient Na^+^ and a delayed rectifier K^+^ along with the leak conductance, while the model producing purely graded signals contained delayed rectifier K^+^ and leak conductances. The dynamics of the membrane potential is governed by a set of activation and inactivation variables *m*, *h and n* with the current balance equation,
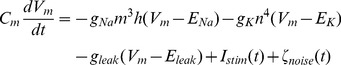
(1)*C_m_* is the membrane capacitance, 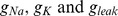 are the conductance of the Na^+^, K^+^ and leak channels respectively, 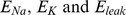 are the respective reversal potentials, 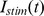 is a time dependent current stimulus and 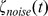 is the input (extrinsic) stimulus noise current. 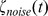 is zero for no input noise simulations. The variables *m*, *h and n* follow first order kinetics of the form 
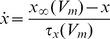
, where 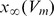 is the steady-state (in) activation function and 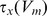 is the voltage-dependent time constant. The model was driven using a time dependent current – 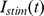, a 300 Hz Gaussian white noise, filtered using a 40^th^ order Butterworth filter. The voltage resonant frequency of the squid axon model can vary between 100 Hz at 10°C to 250 Hz at 20°C [75]. Therefore, we selected the input cut-off frequency at 300 Hz that is slightly more than the output 3 dB cut-off frequency encompassing the frequency response expected out of an underdamped second-order response (see Figure 3 in Guttman et al. [75]). The mean and the standard deviations of the stimulus were varied in the range 1–10 µ*A*/*cm*^2^, enabling comparison to earlier work studying channel noise and its effects on information rates [76]. The stimulus was presented for 1 second and each set of simulations consisted of 60 such trials. 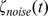 is an unfiltered broad-band Gaussian white noise with,
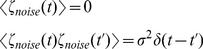
(2)where noise variance is computed using
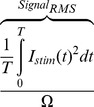
(3)Ω denotes the signal-to-noise ratio (SNR).

All Gaussian random numbers were generated using the Marsaglia's Ziggurat algorithm [77]; uniform random numbers were generated using Mersenne Twister algorithm [78]. Deterministic equations were integrated using the Euler-algorithm while stochastic differential equations were integrated using the Euler-Maruyama method, both with a step size of 10 µ*s*. Parameter values are given in Table S1.

### Gillespie algorithm for simulating channel noise

Our simulations incorporate Na^+^ and the K^+^ voltage-gated ion channels without cooperativity (Figure S8) so that the state transition matrix evolves according to a Markov process [79], [80]. We track the numbers of channels that were either closed or open [79] using the Gillespie algorithm [81]. The Na^+^ and the K^+^ channels had 13 states with 28 possible transitions among these states −20 transitions for the Na^+^ channels and 8 for the K^+^ channels. As an example, in time interval *δt*, the probability that the K^+^ channel remains in state *k* is 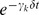, where *γ_k_* depicts the sum of all transition rates from state *k* to any possible successive state. During the interval *δt* no other ion channel changes its state such that the probability of the ion channels remaining in the same state in the time interval *δt* is 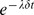,
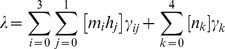
(4)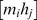 is the number of Na^+^ voltage-gated ion channels in state [*i*, *j*], [*n_k_*] is the number of K^+^ voltage-gated ion channels in state [*k*], *γ_ij_* is the total transition rate from state 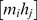 and *γ_k_* is the total transition rate from state 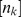. The transition rate 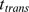 for a particular ion channel state is chosen by drawing a pseudo-random number *r*_1_ from a uniform distribution [0, 1] and defining *t_trans_* as 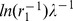. The Gillespie algorithm then selects which of the 28 possible transitions occur in the time interval *t_trans_* [79], [81]. The conditional probability of a particular transition *j* that occurs in the time interval *δt* is given by,
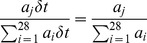
(5)Here, *a_j_* is the product of transition rate associated with transition *j* and the number of channels in the original state of that transition. The denominator in Eqn. (5) is equal to *λ*. The particular transition rate is selected by drawing a random number *r*_2_ from the uniform distribution [0, 1] and fixing *ψ* as,
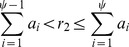
(6)The number of voltage-gated ion channels in each state was updated and the membrane potential calculated. An identical algorithm was used for the channel noise in the compartment containing only K^+^ voltage-gated channels.

### Information-theoretic and linear systems analyses

Both information-theoretic and linear system analysis are a common place in neuroscience [82], but before providing a detailed exposition for each of these methods, we justify our use of them. The channel capacity for a Gaussian channel [25], [56] allows us to place an upper bound on the Shannon information encoded in the generator potentials under the assumption of an additive Gaussian noise. On the other hand, the “direct method” [26] is a minimal assumption method to derive an estimate of the reduction in entropy per unit time per spike. Although these two calculations enable us to quantify the information loss separately within each domain (graded and spiking), a more appropriate comparison would employ the same metric permitting direct comparison between domains. The Wiener filter [9], [56] permits such a comparison, allowing us to test the fidelity of both the analog and the pulsatile signals using identical linear optimal filtering, giving a lower bound on the information present in the response (e.g. to linearly decode the input stimulus). Thus, if inputs were linearly mapped onto outputs then the information rates from “direct method” and the “Wiener filter” analysis would be identical [82]. The lower our reconstruction error the better our generative model of the output is.

### Information rates for spiking neuron models

There are several methods that have been used to quantify information rates in spiking neurons. These include histogram based “direct method” [26], context-tree Markov Chain Monte Carlo (MCMC) [83], metric space method [84], binless method [85], compression entropy [6], among others. We have used the widely employed “direct method” to measure the entropy of the responses, primarily due to its simplicity and the separation of mutual information into separate terms capturing variability (spike train entropy) and reproducibility (noise entropy) [26]. The spike train entropy quantifies the variability of the spike train across time. The noise entropy on the other hand, measured the reproducibility of the spike train across trials. These quantities were dependent upon the temporal resolution with which the spikes were sampled, Δ*t* and the size of time window, *T*. We present a different stimulus current in each subsequent trial (unfrozen noise) to calculate the spike train entropy, while using presentations of the same stimulus current in each subsequent trial (frozen noise) to calculate the noise correlation. We divided the spike train to form K-letter words (K = 2, 4, 6, 8, 12, 16, 24, 32, 48 or 64), where *K* = *T*/Δ*t*. We used the responses from the unfrozen noise session, to estimate the probability of occurrence of particular word, *P*(*W*). We estimated the total entropy as,
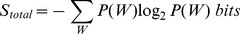
(7)We estimated the probability distribution of each word at specified time durations, *t* so as to obtain *P*(*W*|*t*). Entropy estimates were then calculated from these distributions and the average of the distributions at all times were computed to yield the noise entropy as,
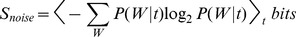
(8)〈〉 indicates average over time. The information was then computed as,
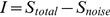
(9)The spike train entropy and the conditional noise entropy diverge in the limit of Δ*τ*→0, their difference converges to the true finite information rate in this limit [26]. Therefore, we used bias correction methods such that the estimation of entropy was less prone to sampling errors [86]. Using Δt = 1 *ms*, we varied the spike trains to form words of different lengths. Using these entropy estimates, we extrapolated to infinite word length from four most linear values of the curve of entropy against the inverse of word length.

### Information rates for nonspiking neuron models

We used an upper-bound method to calculate the maximum information transferable by the nonspiking responses [25], [56]. This method assumes that the neuronal response and the neuronal noise had independent Gaussian probability distributions in the frequency domain and the noise was additive in nature. In the presence of additive non-Gaussian noise such a method provides us with an upper bound on the channel capacity that is dependent on the entropy power of the non-Gaussian noise distribution [25], [87], [88]. We defined the stimulus *S* as the mean neuronal response obtained from a frozen noise experiment. The noise in each trial was calculated by removing the average response from the individual responses *R_i_*. Owing to Gaussian assumptions, it required enough data to estimate the mean and variance of the Gaussian probabilities. The actual information might be lower than this bound because a Gaussian distribution has the highest entropy for a given variance. In our simulations, both the response and the noise had an approximately Gaussian distribution. We obtained the mean response power spectrum and the noise power spectrum using the multi-taper spectral estimator and computed their ratio to be the signal-to-noise ratio (SNR) [29]. This is then used to compute the information for the Gaussian channel as,
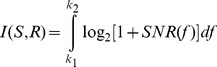
(10)For our simulations, the limits of the integral were taken from *k*_1_ = 0 Hz to *k*_2_ = 300 Hz. The integral was evaluated using trapezoidal rule.

### Stimulus reconstruction

We performed stimulus reconstruction to test how noise affects the coherence of a linear system [9], [56]. The method involved finding a linear temporal filter to minimize the difference between the real and the reconstructed stimulus. We followed Haag and Borst [89] in the derivation of this filter using Gaussian unfrozen noise as the stimulus set. We used 60 trials that consisted of 1 second period of unfrozen white noise *s_i_*(*t*) to obtain the spike trains *r_i_*(*t*) in the form of 1's and 0's with 10 µ*s* resolution. These time domain signals were Fourier-transformed to obtain complex functions *S_i_*(*f*) and *R_i_*(*f*). Two filters were obtained, either by normalizing the cross-power spectral densities (CPSD) of the stimulus and the spike response by the stimulus power spectral density (PSD) (*forward filter*) or the spike power spectral density (*reverse filter*) as demonstrated below, with angle brackets (< >) indicating averages over trials,
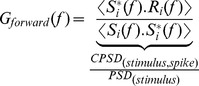
(11)
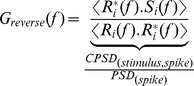
(12)Using the reverse filter, we estimated the stimulus, 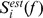 as the product between *R_i_*(*f*) and *G_reverse_*(*f*),
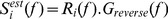
(13)The quality of the estimate was evaluated by computing a filter between the original stimulus and the reconstructed stimulus; this is simply the coherence function (*γ*^2^(f)) as shown below,
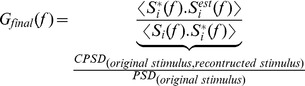
(14)
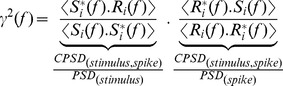
(15)The coherence results have been cross-validated using a 65–35 split between the training set and the test set i.e., we used the first 65% of the trials to calculate the reverse filter and then checked its validity on the next 35% of the trials by computing the final filter (*G*_fnal_(*f*)) or the actual coherence (*γ*^2^(*f*)).

Reconstruction quality was measured using two metrics. First, normalized root mean squared error (nRMSE) between the original stimulus and the reconstructed stimulus was calculated as,
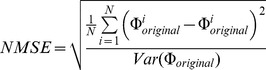
(16)A nRMSE value that tends towards zero represents perfect reconstruction. Second, we calculated a coherence based information rate where a higher value indicates better reconstruction,
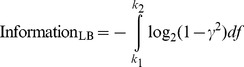
(17)

### Calculation of energy

Energy consumption in our model is defined as the amount of ATP expended during the encoding of the band-limited stimulus current. The Na^+^–K^+^ pump hydrolyses one ATP molecule for three Na^+^ ions extruded out and two K^+^ ions imported into the cell [11]. We determined the total K^+^ current by separating the leak current into a K^+^ permeable leak current and adding it to the delayed rectifier K^+^ current. We computed the number of K^+^ ions by integrating the area under the total K^+^ current curve for the duration of stimulus presentation. In order to derive the energy consumption we calculated the number of ATP molecules used by multiplying the total K^+^ charge by N_A_/(2F), where N_A_ is the Avogadro's constant and F is the Faraday's constant.

## Supporting Information

Figure S1 **Input stimulus statistics affect the reliability of action potentials generated by the spiking neuron model.** A. An example of a low mean, high standard deviation current stimulus (upper trace) and the action potentials evoked in the spiking neuron model with stochastic voltage-gated Na^+^ channels and stochastic voltage-gated K^+^ channels (middle trace). A raster plot (lower graph) of the action potentials evoked by presenting the same current stimulus 60 times. B. An example of a high mean, low standard deviation current stimulus (upper trace) and the action potentials evoked in the spiking neuron model with stochastic voltage-gated Na^+^ channels and stochastic voltage-gated K^+^ channels (middle trace). A raster plot (lower graph) of the action potentials evoked by presenting the same current stimulus 60 times.
(EPS)

Figure S2 **The effect of voltage-gated Na^+^ or K^+^ channel noise on action potentials generated by the spiking neuron model.** A. An example of a low mean, high standard deviation current stimulus (upper trace) and the action potentials evoked in the spiking neuron model with stochastic voltage-gated Na^+^ channels and deterministic voltage-gated K^+^ channels (middle trace). A raster plot (lower graph) of the action potentials evoked by presenting the same current stimulus 60 times. B. An example of the action potentials evoked in the spiking neuron model with deterministic voltage-gated Na^+^ channels and stochastic voltage-gated K^+^ channels (middle trace) in response the same current stimulus (upper trace) as in A. A raster plot (lower graph) of the action potentials evoked by presenting the same current stimulus 60 times.
(EPS)

Figure S3 **Linear decoding performance using action potentials, generator potentials and graded voltage responses.** A. The blue trace represents the input stimulus current while the red trace represents linear reconstruction based entirely on the spiking response. The current input had a mean and standard deviation set at 0 µA/cm^2^ and 5 µA/cm^2^ respectively. B. The blue trace represents the input stimulus current and the red trace represents linear reconstruction based entirely on the pseudo-analog response. C. The blue trace represents the input stimulus current while the red trace represents linear reconstruction based entirely on the graded response. D. Normalized mean squared error (nRMSE) between the original and the reconstructed input. The mean and standard deviation of the inputs were sampled from N(0, 2), N(0, 5), N(0, 10), N(5, 5), N(10, 5) *and* N(10, 10). E. Coherence based mutual information for the inputs in D.
(EPS)

Figure S4 **The effect of extrinsic noise on action potentials generated by the spiking neuron model.** A. Total entropy, B. noise entropy, and C. mutual information of the spike trains generated in response to white noise current stimuli with different means and standard deviations. The Signal-to-Noise ratio (SNR) of the input was fixed at 2.
(EPS)

Figure S5 **Extrinsic noise reduces mutual information in spike trains, pseudo-generator potentials and graded potentials.** A. Introduction of extrinsic noise with SNR = 2 causes a 40% decrease in mutual information in the spiking responses. B. Similarly, there is a decrease of up to 60% in the pseudo-generator potentials in response to inputs with SNR = 2. C. Over a wide variety of inputs, the mutual information is decreased up to 70% in the graded potentials with the introduction of extrinsic noise.
(EPS)

Figure S6 **The numbers of open K^+^ channels and the membrane potential range determines the energy consumption of pseudo-generator potentials.** A. Joint kernel density estimates of open K^+^ channels and the membrane potential in response to low mean, low standard deviation stimulus, and B. low mean, high standard deviation stimulus. C. Joint kernel density estimates of open K^+^ channels and the membrane potential in response to high mean, low standard deviation stimulus, and D. high mean, high standard deviation stimulus.
(PDF)

Figure S7 **The numbers of open K^+^ channels and the membrane potential range determines the energy consumption of graded potentials.** A. Joint kernel density estimates of open K^+^ channels and the membrane potential in response to low mean, low standard deviation stimulus, and B. low mean, high standard deviation stimulus. C. Joint kernel density estimates of open K^+^ channels and the membrane potential in response to high mean, low standard deviation stimulus, and D. high mean, high standard deviation stimulus.
(PDF)

Figure S8 **The gating scheme for the voltage-gated ion channels.** A. State transition diagram for Na^+^ channel. B. State transition diagram for K^+^ channel.
(EPS)

Table S1 **Parameters for the stochastic Hodgkin-Huxley model.**
(DOCX)
