# Supplementary figures and images for: Consequences of Converting Graded to Action Potentials upon Neural Information Coding and Energy Efficiency
Source: PLoS Comput Biol. 2014 Jan 23;10(1):e1003439. doi: 10.1371/journal.pcbi.1003439 (PMC3900385; doi:10.1371/journal.pcbi.1003439)

**A**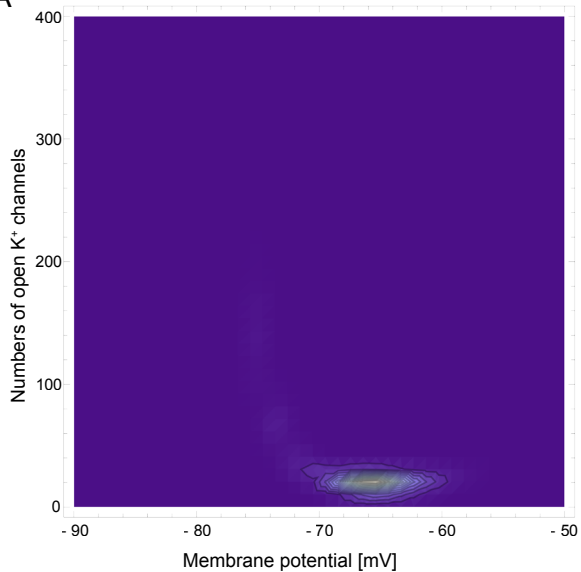**B**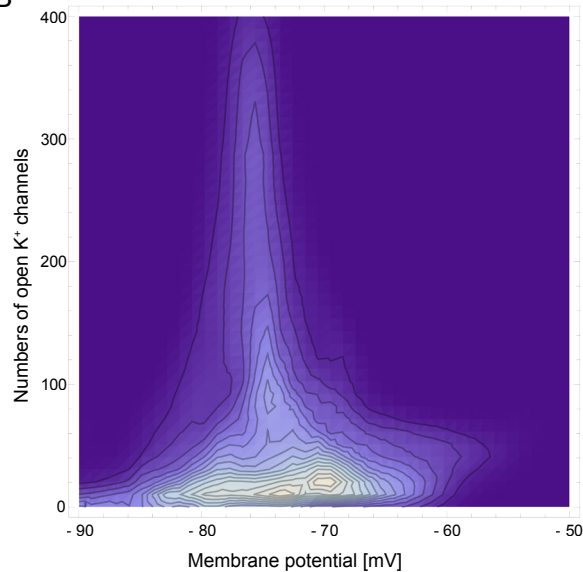**C**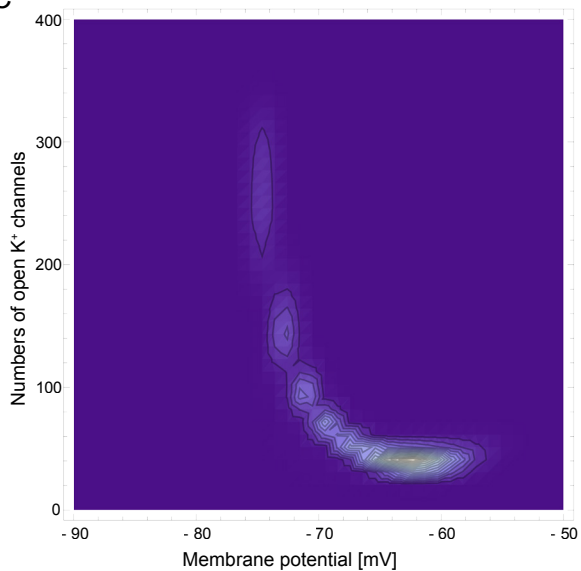**D**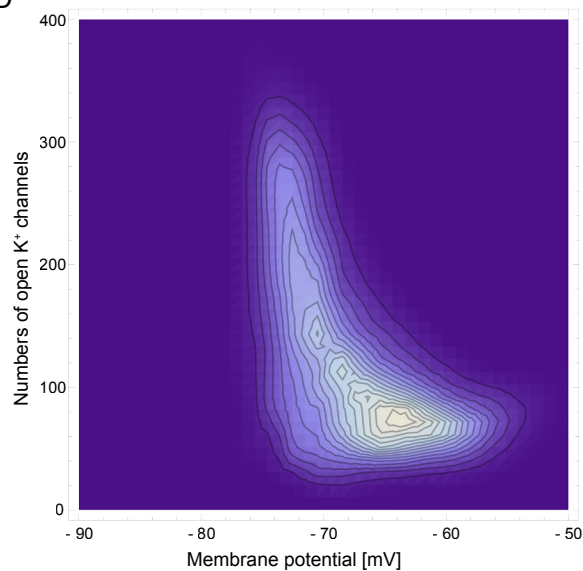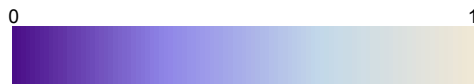

Supplement: Figure S6 — The numbers of open K+ channels and the membrane potential range determines the energy consumption of pseudo-generator potentials. A. Joint kernel density estimates of open K+ channels and the membrane potential in response to low mean, low standard deviation stimulus, and B. low mean, high standard deviation stimulus. C. Joint kernel density estimates of open K+ channels and the membrane potential in response to high mean, low standard deviation stimulus, and D. high mean, high standard deviation stimulus. (PDF) [file pcbi.1003439.s006.pdf]

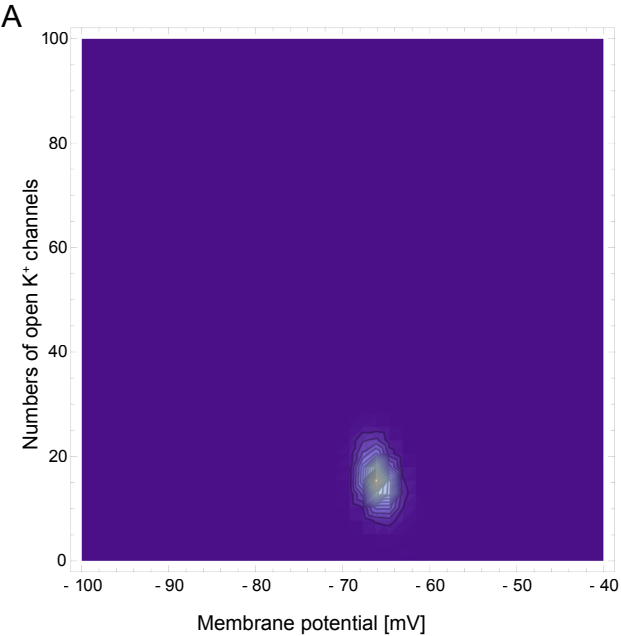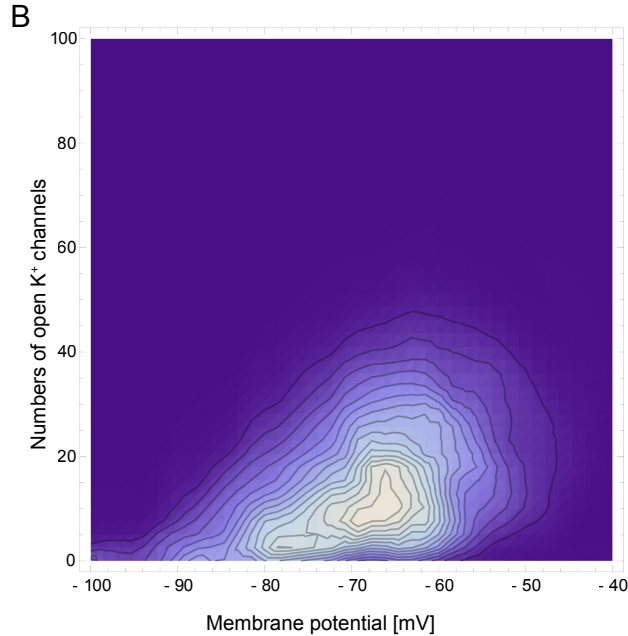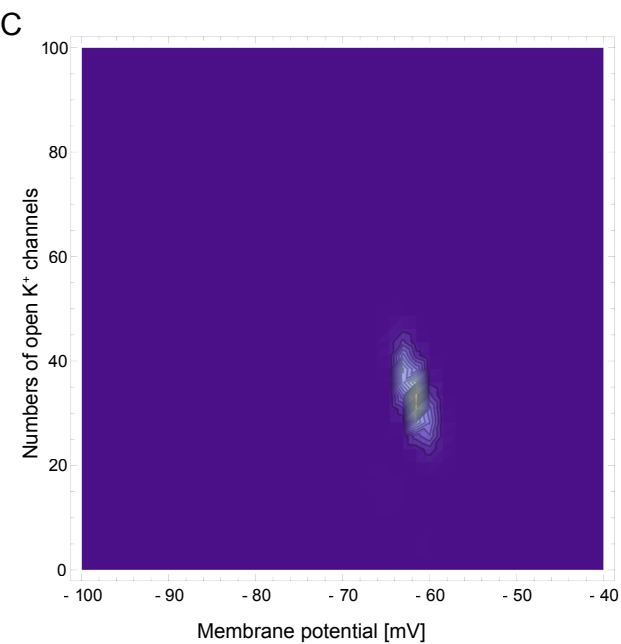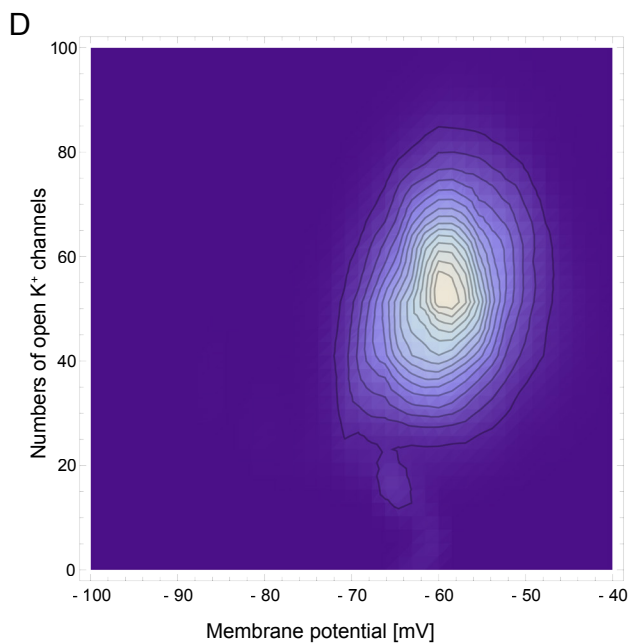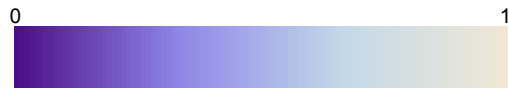

Supplement: Figure S7 — The numbers of open K+ channels and the membrane potential range determines the energy consumption of graded potentials. A. Joint kernel density estimates of open K+ channels and the membrane potential in response to low mean, low standard deviation stimulus, and B. low mean, high standard deviation stimulus. C. Joint kernel density estimates of open K+ channels and the membrane potential in response to high mean, low standard deviation stimulus, and D. high mean, high standard deviation stimulus. (PDF) [file pcbi.1003439.s007.pdf]
